# Comprehensive resource for transcription readthrough events in healthy human tissues

**DOI:** 10.1038/s41597-025-05557-w

**Published:** 2025-07-10

**Authors:** Yang Mei, Ziqi Cheng, Yueqi Lu, Shiyi Wu, Xi Chen

**Affiliations:** 1 College of Plant Protection, Jilin Agricultural University, Changchun, 130118 China; 2 Yangtze Delta Region Institute (Quzhou), University of Electronic Science and Technology of China, Quzhou, 324000 China; 3 College of Animal Science and Technology, Jilin Agricultural University, Changchun, 130118 China; 4 Department of Clinical Laboratory, The Quzhou Affiliated Hospital of Wenzhou Medical University, Quzhou People’s Hospital, Quzhou, 324000 China

**Keywords:** Gene expression, Gene regulation, Predictive markers

## Abstract

Transcription readthrough occurs when RNA polymerase bypasses canonical termination sites, producing elongated RNA molecules called readthrough (RT) transcripts or downstream of gene (DoG) transcripts. Although RT transcripts have been implicated in stress responses and pathological states, their roles in healthy human tissues are poorly understood. This study collected and analyzed RT events across 43 healthy human tissues, identifying 75,248 RT events from 35,720 transcripts across 11,692 genes. The dataset encompasses the sequences, locations, expression profiles, and comprehensive annotation information of corresponding genes for RT transcripts. It provides a thorough elucidation of RT transcriptomics and its significance in gene regulation, offering a wealth of benchmark data to facilitate further research on RT transcripts.

## Background & Summary

Transcription termination is a critical regulatory step in gene expression, wherein RNA polymerase ceases RNA synthesis and dissociates from the DNA template upon completing transcription^1^. However, under specific conditions, the transcription machinery may fail to recognize termination signals, resulting in transcription extending beyond the defined gene boundaries—a phenomenon termed transcription readthrough (TRT)^2–5^. This process generates elongated RNA molecules known as readthrough (RT) transcripts or downstream of gene (DoG) transcripts, which have been observed under various stress conditions^6^, including hyperosmotic stress, heat shock^7^, oxidative stress^1,2^, hypoxia^8,9^, viral infections^5,10,11^, and cancer^4,12–15^. These transcripts have been implicated in maintaining chromatin structure and potentially modulating gene expression^2,3,16^. Furthermore, studies have shown that the three-dimensional organization of chromatin not only affects the initiation and extension of transcription but also plays a significant role in transcription termination. Changes in chromatin structure can influence the behavior of RNA polymerase II, thereby regulating the efficiency and precision of transcription termination^17,18^.

TRT disrupts gene regulatory networks by extending into downstream genomic regions, potentially triggering unintended transcription of neighboring genes without promoter activation^19^. This process can also result in functional antisense RNAs that repress gene expression or in the formation of circular RNAs from downstream exons^4^. Furthermore, RT events may produce RNA chimeras through intergenic exon splicing, some of which are linked to tumor proliferation and cancer survival^20^. These findings underscore the multifaceted roles of TRT in both normal cellular processes and disease pathogenesis^1,4,8,9,21^.

Recent evidence demonstrates that RT transcripts are not confined to stress responses or pathological conditions but are also prevalent in healthy human tissues^22^. This widespread occurrence suggests that RT transcripts may play vital physiological roles in maintaining cellular homeostasis^22^. However, systematic large-scale investigations into TRT differences between healthy and diseased tissues remain limited. Previous research has primarily targeted cancer-specific chimeric RNAs and the development of specialized disease-focused databases^21,23,24^, the broader biological implications of RT transcripts, beyond chimeric RNA formation, remain insufficiently explored. Moreover, the current state of TRT research is characterized by fragmented and inconsistent data, with no dedicated platform to support systematic analyses. To address this gap, we generate the comprehensive dataset for TRT in healthy human tissues and developed the online platform (hhrtBase, http://www.hhrtbase.com/). This platform serves as a comprehensive reference, offering an integrated platform for browsing, downloading, and analyzing RT data across various samples. By enabling systematic comparisons, hhrtBase aims to elucidate the functional significance of transcription RT in normal physiology and disease, advancing its applications in biomedical research.

The dataset presented in this study, comprising 75,248 TRT events from 11,692 genes in 43 healthy human tissues. By offering a systematic catalog of TRT events, the dataset enables researchers to investigate the prevalence, distribution, and functional implications of RT transcripts in normal physiology. For example, researchers can use this dataset to identify tissue-specific TRT patterns, correlate TRT events with gene expression profiles, or explore associations between RT transcripts and chromatin organization. Such analyses could reveal novel regulatory mechanisms underlying cellular homeostasis or identify potential biomarkers for physiological states. The dataset’s utility extends to comparative studies between healthy and diseased tissues. For instance, researchers can compare this dataset with cancer TRT data to identify aberrant RT events associated with oncogenesis or tumor progression. Additionally, the dataset supports studies on the evolutionary conservation of TRT events, the impact of genetic variants on RT propensity, and the role of RT transcripts in shaping the non-coding RNA landscape. By providing a robust, curated reference of TRT events in healthy human tissues, this dataset serves as a foundational resource for hypothesis-driven research, enabling scientists to address fundamental questions in gene regulation, chromatin dynamics, and disease biology.

## Data Summary

Analysis of 2,759 RNA-seq samples from 43 tissues revealed 75,248 RT events derived from 11,692 genes. The lengths of these RT transcripts varied significantly, ranging from 2,001 base pairs to over 177,501 kilobases (kb) beyond the annotated gene boundaries, with a median length of approximately 7.7 kb. Notably, some RT events exhibited extraordinary extensions exceeding 177 kb (ENSG00000256499), particularly in tissues such as the artery (aorta) and artery (tibial).

**Fig. 1:**
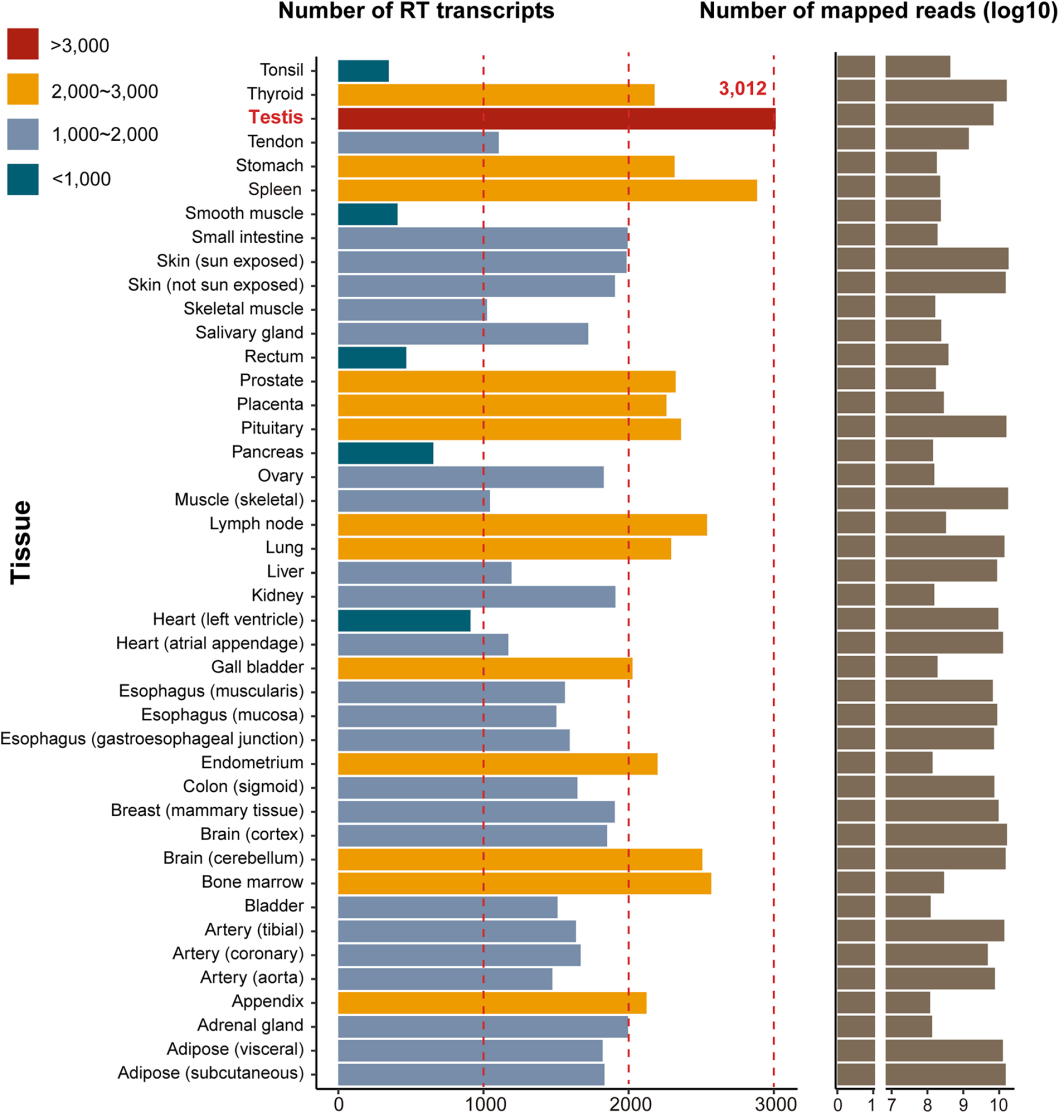
Tissue distribution of RT transcripts in 43 tissues. The first bar chart illustrates the total number of readthrough (RT) transcripts identified in each of the 43 human tissues analyzed. Tissues are categorized based on the number of RT transcripts: >3,000 (red), 2,000–3,000 (yellow), 1,000–2,000 (blue), and <1,000 (teal). The second bar chart displays the logarithm of the total number of mapped reads for each tissue.

The distribution of RT transcripts across 43 tissues revealed variability (Fig. 1). Testis exhibited the highest numbers of RT transcripts, with 3,012 transcripts. Other tissues, including the thyroid, stomach, spleen, prostate, placenta, pituitary, lymph node, lung, gall bladder, endometrium, brain (cerebellum), bone marrow, and appendix, demonstrated moderately elevated RT transcript counts, ranging between 2,000 and 3,000. Most tissues exhibited RT transcripts ranging from 1,000 to 2,000. Conversely, tissues like the tonsil, smooth muscle, rectum, pancreas, and heart (left ventricle) displayed fewer than 1,000 RT transcripts. These differences reflect variations in data volume across tissues. However, whether they indicate actual differences in TRT among tissues requires further in-depth analysis by researchers, including examination of individual samples and expression profiles.

### Analysis example: expression patterns of RT transcripts across tissues

**Fig. 2:**
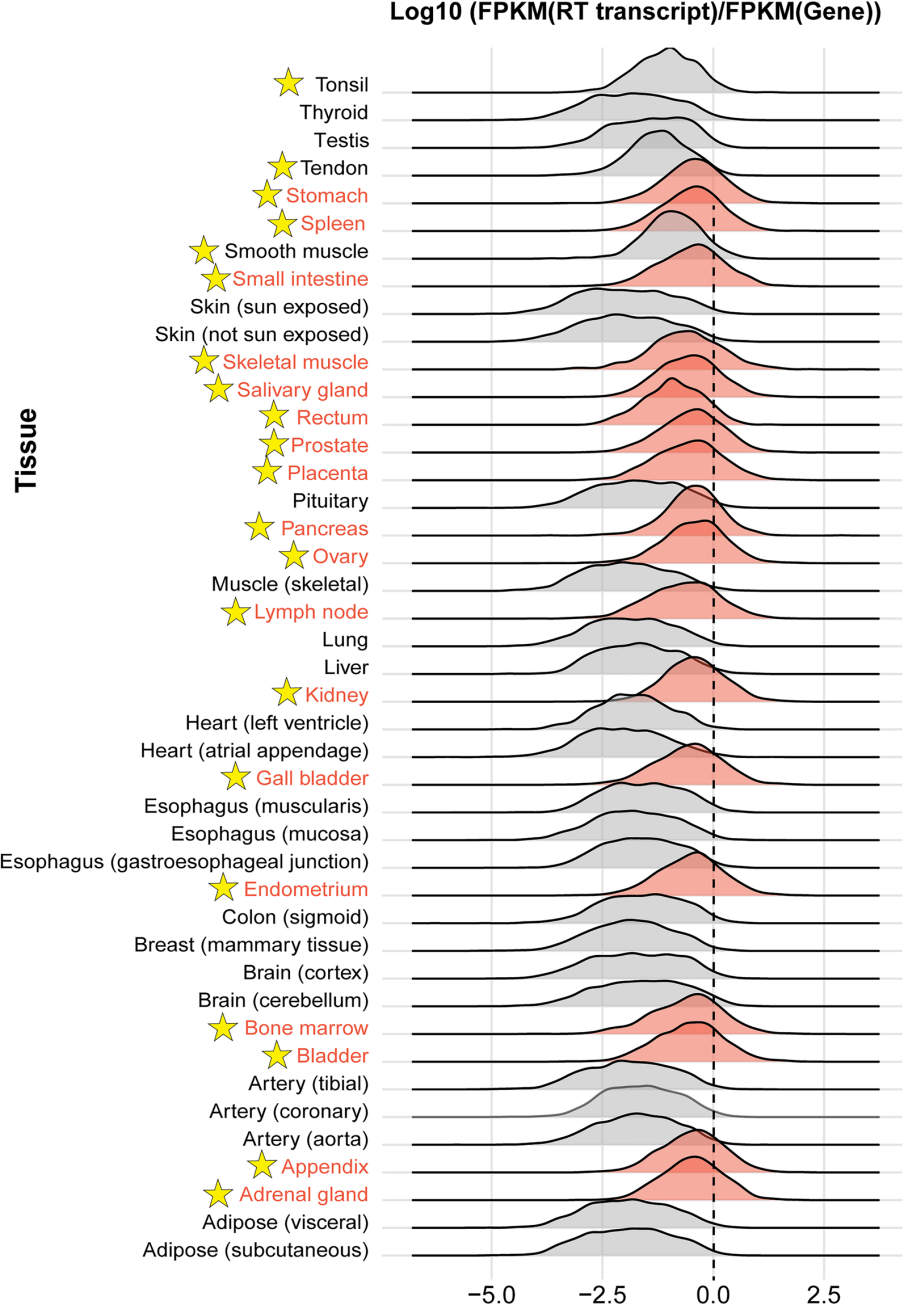
Tissue-specific expression patterns of RT transcripts relative to their corresponding genes. The distribution of the expression ratio between RT transcripts and their corresponding genes, represented as log10(FPKM (RT transcript)/FPKM(Gene)), is shown for 43 tissues. The dashed vertical line at 0 indicates equal expression levels of RT transcripts and their parent genes. Negative values reflect the lower RT transcript expression relative to their genes, while positive values indicate higher RT transcript expression. The data from tissues marked with a star were derived from stranded RNA-seq libraries, while those without a marker came from unstranded RNA-seq libraries.

The expression ratio between RT transcripts and their corresponding genes revealed pronounced tissue-specific differences in RT transcript expression (Fig. 2). It should be noted that genes lacking RT transcripts are not displayed in the figure. Therefore, the figure illustrates the expression relationship between genes with RT transcripts and their corresponding RT transcripts across different tissues, rather than depicting the expression patterns of all genes in these tissues. We have accordingly labeled the two distinct RNA-seq approaches (stranded vs. unstranded) to facilitate comparative analysis by researchers (Fig. 2).

In most tissues, the distribution of expression ratios peaked below 0, indicating that RT transcripts are generally expressed at lower levels than their parent genes. However, the degree of this difference varied considerably across tissues. Notably, tissues such as the stomach, spleen, and small intestine (highlighted in red in Fig. 2) exhibited distributions closer to 0, suggesting that RT transcripts in these tissues are expressed at levels comparable to their corresponding genes. This observation may reflect the functional importance of RT transcripts in these transcriptionally dynamic tissues, where they might play roles in chromatin remodeling, the generation of alternative RNA isoforms, or other regulatory processes.

Conversely, tissues such as the testis, lung, and liver (highlighted in grey in Fig. 2) exhibited expression distributions with peaks significantly below −2, indicating that RT transcripts are expressed at markedly lower levels relative to their parent genes. This pattern suggests stringent regulation of TRT in these tissues, likely minimizing its impact on downstream genes and chromatin structure. The breadth of these distributions also varied across tissues. For example, the liver and thyroid displayed broader distributions, reflecting heterogeneity in RT transcript expression, with some transcripts achieving relatively high expression levels while others remained low. In contrast, tissues such as the kidney and pancreas exhibited narrower distributions, indicating more consistent and uniform RT transcript expression relative to their parent genes. These findings highlight substantial variability in RT transcript expression patterns across tissues, underscore the diverse roles of RT transcripts in gene regulation and their contribution to maintaining tissue-specific transcriptional equilibrium and provides a framework for deeper investigation into its biological significance.

## Methods

### Data collection

A comprehensive set of publicly available RNA-seq datasets and TRT data representing healthy human tissues was collected from National Center for Biotechnology Information (NCBI) and relevant published literature (10.6084/m9.figshare.24848265.v3)^22,25^ (Supplementary Table S1). The human reference genome (GRCh38.p13) and corresponding gene annotation files (version 37) were obtained from the GENCODE database^26^.

### Data analysis

The raw sequencing data underwent a rigorous quality control process to ensure reliability. Initially, the quality of raw RNA-seq reads was assessed using FastQC v0.12.1 (http://www.bioinformatics.babraham.ac.uk/projects/fastqc/), and the quality report was compiled with MultiQC v1.9^27^. Low-quality bases and adapter sequences were removed using Trimmomatic v0.39^28^, employing default parameters alongside additional trimming thresholds for precision.

Subsequently, the high-quality reads were aligned to the reference genome using STAR v2.7.9a^29^. After alignment, TRT events were identified through ARTDeco^30^ with default parameters.

For the publicly available TRT data, all were derived from GTEx samples (10.6084/m9.figshare.24848265.v3). Since GTEx samples were profiled using non-stranded RNAseq libraries, we filtered the results to report only entries that did not overlap with genes on the opposite strand, using the intersect function from bedtools (v2.30.0)^31^.

Additionally, we excluded the RT transcripts of non-expressed genes in each specific tissue. Expressed genes are defined as those with FPKM > 1 in at least 25% of the samples within a specific tissue.

For all identified TRT events, we used subseq function from seqtk (1.4-r122) (https://github.com/lh3/seqtk) to extract their sequences based on their positional information and the specific version of the genome downloaded above. The gene annotation information was extracted based on the Ensembl Gene ID from the following URL: https://grch37.ensembl.org/index.html and selected the median expression level across all samples as the data for plotting on the online platform.

## Data Records

We have publicly shared the dataset on Figshare^32^ (10.6084/m9.figshare.28974116) in CSV format, containing the following information for each Downstream-of-Gene (DoG) transcript:

**Genomic localization details** of both the gene *(chromosome, start_position, end_position, strand)* and the DoG transcript (*chromosome (DoG), dog_start_position, dog_end_position, strand (DoG)*).

**Gene annotations** (*gene_id, Symbol, Synonym, Description*) including functional descriptions and identifiers.

**Sequence information** (*sequence*) of the DoG transcript.

**Tissues information** (*tissue*) of the DoG transcript.

**Average expression levels** (*mean-geneFPKM for the gene, mean-dogFPKM for the DoG transcript*) across samples.

**All expression data** with per-sample values (*all-geneFPKM, all-dogFPKM*) and corresponding sample_ids.

**Unique identifiers** (*DOG for the DoG transcript, gene_id for the associated gene*).

## Technical Validation

To systematically identify TRT events, we implemented a robust analytical pipeline using STAR-aligned BAM files and ARTDeco, a computational framework specifically designed for transcriptional readthrough characterization. ARTDeco employs a sliding window algorithm to detect continuous RNA-seq read coverage extending beyond the 3′ end of gene annotations by at least a default or user-defined minimum threshold. Transcriptional readthrough candidates were defined as regions meeting coverage thresholds across consecutive windows. To ensure analytical rigor, all downstream analyses exclusively utilized uniquely mapped reads identified through HOMER’s tools (v4.11)^33^, eliminating ambiguities from multi-mapped reads.

To maximize the reliability of TRT predictions, we implemented stringent quality control standards throughout all stages of data processing. To effectively mitigate batch effects, RNA-seq data were rigorously curated based on both tissue type and sequencing project criteria: for samples of the same tissue type, only those within the same study project were included, thereby completely avoiding interference caused by cross-project data integration. Recognizing that bidirectional transcriptional noise could confound TRT detection, we restricted analyses to strand-specific RNA-seq libraries. This critical filtering step enabled unambiguous assignment of transcriptional directionality, excluding signals from antisense transcription or overlapping genes on the reverse strand. Raw RNA-seq reads were first evaluated with FastQC, followed by comprehensive quality report generation using MultiQC. Subsequently, Trimmomatic was applied to filter out low-quality bases and adapter sequences, utilizing both default parameters and customized trimming thresholds to enhance processing accuracy.

For public TRT datasets derived from non-stranded GTEx libraries with ARTDeco, we implemented an additional validation layer using BEDTools (v2.30.0). Putative readthrough regions intersecting genes on the reverse strand were systematically excluded via bedtools intersect. Alignment and TRT detection tools between our pipeline and published TRT data were rigorously harmonized (STAR alignment, identical genome build, ARTDeco for prediction TRT). This methodological congruence enabled direct comparison while maintaining internal validity.

This multi-tiered quality control framework—spanning experimental design constraints, computational filtering, and directional specificity validation-ensured high-confidence TRT identification while addressing inherent limitations of transcriptional readthrough analyses in complex eukaryotic genomes.

## Usage Notes

All data is directly downloadable from Figshare. Additionally, we have integrated these resources into our custom-developed online platform (http://www.hhrtbase.com/) that incorporates various analytical tools, providing convenient access for browsing, downloading, and utilization.

## Supplementary information

The information of samples

## Data Availability

No custom code was used. Software tools used for processing are mentioned in the Methods and Technical Validation sections.
